# Plasma level and expression of visfatin in the porcine hypothalamus during the estrous cycle and early pregnancy

**DOI:** 10.1038/s41598-021-88103-z

**Published:** 2021-04-22

**Authors:** Tadeusz Kaminski, Marta Kiezun, Ewa Zaobidna, Kamil Dobrzyn, Barbara Wasilewska, Ewa Mlyczynska, Edyta Rytelewska, Katarzyna Kisielewska, Marlena Gudelska, Kinga Bors, Grzegorz Kopij, Karolina Szymanska, Barbara Kaminska, Agnieszka Rak, Nina Smolinska

**Affiliations:** 1 Department of Animal Anatomy and Physiology, Faculty of Biology and Biotechnology, University of Warmia and Mazury in Olsztyn, Oczapowskiego St. 1A, 10-719 Olsztyn-Kortowo, Poland; 2 Department of Physiology and Toxicology of Reproduction, Institute of Zoology and Biomedical Research, Jagiellonian University in Krakow, Gronostajowa St. 9, 31–387 Krakow, Poland; 3 Department of Human Physiology and Pathophysiology, School of Medicine, University of Warmia and Mazury in Olsztyn, Warszawska St. 30, 10-082 Olsztyn, Poland

**Keywords:** Reproductive biology, Endocrinology

## Abstract

Visfatin appears to be an energy sensor involved in the regulation of female fertility, which creates a hormonal link integrating the control of energy homeostasis and reproduction. This study evaluates the expression levels of visfatin gene and protein in selected areas of the porcine hypothalamus responsible for gonadotropin-releasing hormone synthesis: the mediobasal hypothalamus (MBH) and preoptic area (POA), and visfatin concentrations in the blood plasma. The tissue samples were harvested from gilts on days 2–3, 10–12, 14–16, and 17–19 of the estrous cycle, and on days 10–11, 12–13, 15–16, 27–28 of pregnancy. Visfatin was localized in the cytoplasm and nucleus of cells creating both studied hypothalamic structures. The study demonstrated that visfatin gene and protein expression in MBH and POA depends on hormonal status related to the phase of the estrous cycle or early pregnancy. Blood plasma concentrations of visfatin during the estrous cycle were higher on days 2–3 in relation to other studied phases of the cycle, while during early pregnancy, the highest visfatin contents were observed on days 12–13. This study demonstrated visfatin expression in the porcine hypothalamus and its dependence on the hormonal milieu related to the estrous cycle and early pregnancy.

## Introduction

Visfatin, also termed nicotinamide phosphoribosyltransferase (NAMPT), was identified in 2005 by Fukuhara et al.^1^. It exists in two forms: the intracellular form, which is a rate-limiting enzyme engaged in nicotinamide adenine dinucleotide (NAD) biosynthesis from nicotinamide and the extracellular form considered as an adipokine. Until now, no visfatin receptor has been identified. On the other hand, it is suggested that visfatin can bind and activate insulin receptor (INSR), and downstream signaling pathways^2^. The adipokine and insulin do not compete for binding to INSR, which implies that these two hormones recognize different regions of the receptor^3^. Apart from the adipose tissue, visfatin was also expressed in tissues creating the hypothalamic-pituitary–gonadal (HPG) axis: in the mouse hypothalamus^4^, in the pituitaries collected from sheep^5^ and mice^4^, in the ovarian follicular cells of humans^6,7^, cows^8^ and mice^4^ as well as bovine corpora lutea^8^. Visfatin was also found in the porcine tissues, including the pituitary and ovary^9^. Plasma and peri-renal adipose tissue NAMPT levels were not different between the two breeds of pigs, fat Meishan and lean Large White, suggesting that this adipokine is not involved in fattening in swine^10^. The occurrence of visfatin mRNA and protein in various tissues suggests that non-adipose tissue may also contribute to serum visfatin levels. Visfatin expression in adipocytes can be affected by hormonal factors such as steroid hormones^11^, tumor necrosis factor α (TNFα)^9,12^, growth hormone (GH)^12^ and dexamethasone^11,12^, while in the human granulosa cells by human chorionic gonadotropin (hCG) and prostaglandin E_2_^6^. It is suggested that visfatin gene expression can be controlled by species-specific regulatory mechanism^9^ and the adipokine concentrations in human adipose tissue are affected by hormonal status related to pregnancy^14^. The circumstances showing that the hormonal milieu typical for pregnancy may affect visfatin production are demonstrated in a study by Mastorakos et al.^15^ indicating the increase in visfatin plasma concentrations with advancing gestational age of women. However, the effect of hormonal changes associated with the individual phases of the estrous cycle and periods of pregnancy on the levels of visfatin transcript and protein has not been analyzed in the hypothalamus.

Visfatin is likely to have pleiotropic properties. It plays a role in the control of energy homeostasis, inflammation, and cell differentiation^16^. Circulating visfatin concentrations correlate positively with body mass index and obesity^12,17^. As an insulin-mimetic hormone, visfatin stimulates glucose uptake in muscle cells and adipocytes^18^ and suppresses glucose release from hepatocytes^19^. Visfatin, like other adipokines^20–23^, could be an energy sensor involved in regulating female fertility. Administration of visfatin during the superovulation of mice increased the developmental competence of oocytes and fertility potential^24^. In women undergoing ovarian stimulation, a correlation was found between visfatin concentration in the ovarian follicular fluid and the number of oocytes retrieved^6^. An effect of visfatin on the ovarian steroidogenesis was also demonstrated^7,8^. It also seems that visfatin plays an important role in the implantation and placentation^18,25^. It is plausible that the action of visfatin during the estrous cycle and early pregnancy (maternal recognition of pregnancy, implantation, placentation) is partly achieved through its influence on the endocrine HPG axis, including the hypothalamus, what has not been investigated so far.

Visfatin may act through the regulation of the hypothalamic structures secreting gonadotropin-releasing hormone (GnRH)—the key factor controlling the pituitary and ovaries. We hypothesized that hypothalamic visfatin expression is dependent on animal hormonal status. Therefore, the goal of the present study was to investigate visfatin gene and protein expression, and its localization in the porcine hypothalamic structures: mediobasal hypothalamus (MBH) and preoptic area (POA) engaged in GnRH generation, and plasma visfatin concentrations during the estrous cycle and early pregnancy.

## Results

### The distribution of visfatin in the porcine hypothalamus

**Figure 1:**
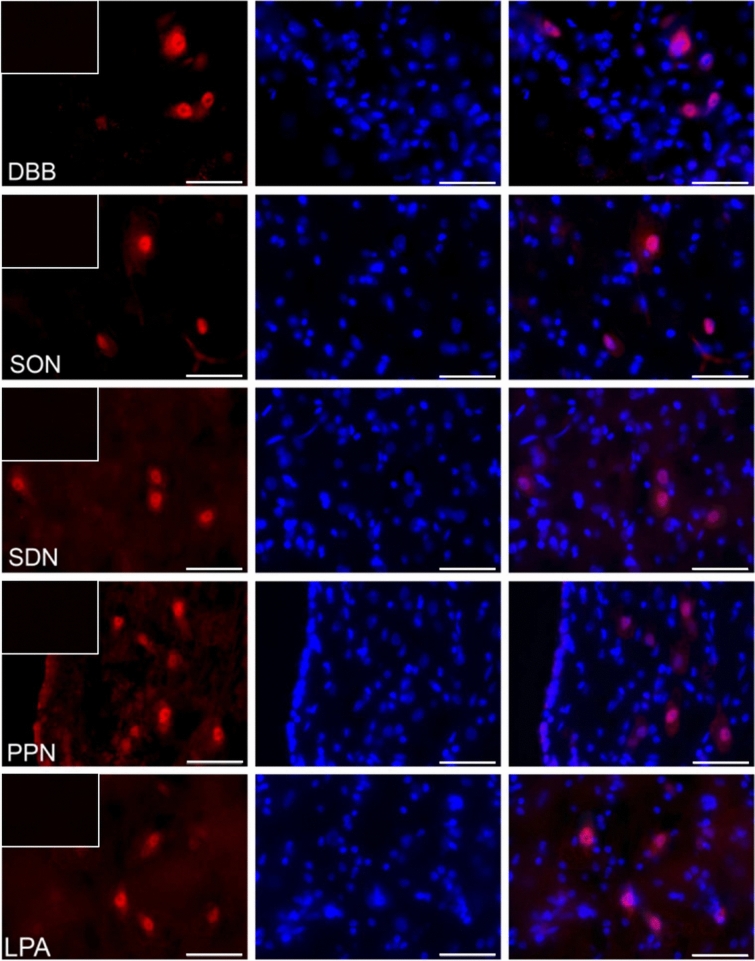

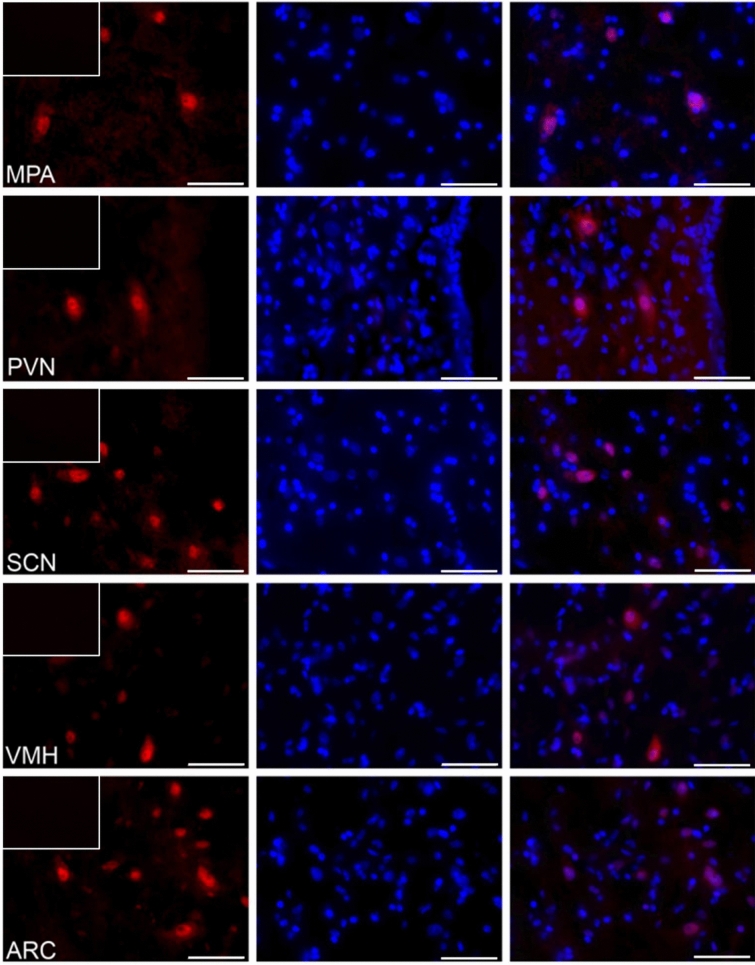
The visfatin localization in the porcine hypothalamus on days 10–12 of the estrous cycle. Immunoreactivity of visfatin was determined by fluorescent immunohistochemistry. Columns from left: first—visfatin expression, visualized by Alexa Fluor 555 as red fluorescence; second—nuclei stained with DAPI, visualized as blue fluorescence; third—merged images of channels (Olympus BX51, Olympus Soft Imaging Solutions, Germany). In the upper left corner of each image in the first column—negative control, where the primary antibody were replaced with non-immunosera. Particular images indicate the immunolocalization of visfatin in the diagonal band of Broca (DBB), supraoptic nucleus (SON), preoptic area (SDN), periventricular nucleus (PPN), lateral (LPA) and medial preoptic area (MPA), paraventricular nucleus (PVN), suprachiasmatic nucleus (SCN), arcuate nucleus (ARC), as well as ventromedial nucleus (VMH). Immunofluorescent staining was repeated on three pigs. Scale bar: 50 μm.

**Figure 2:**
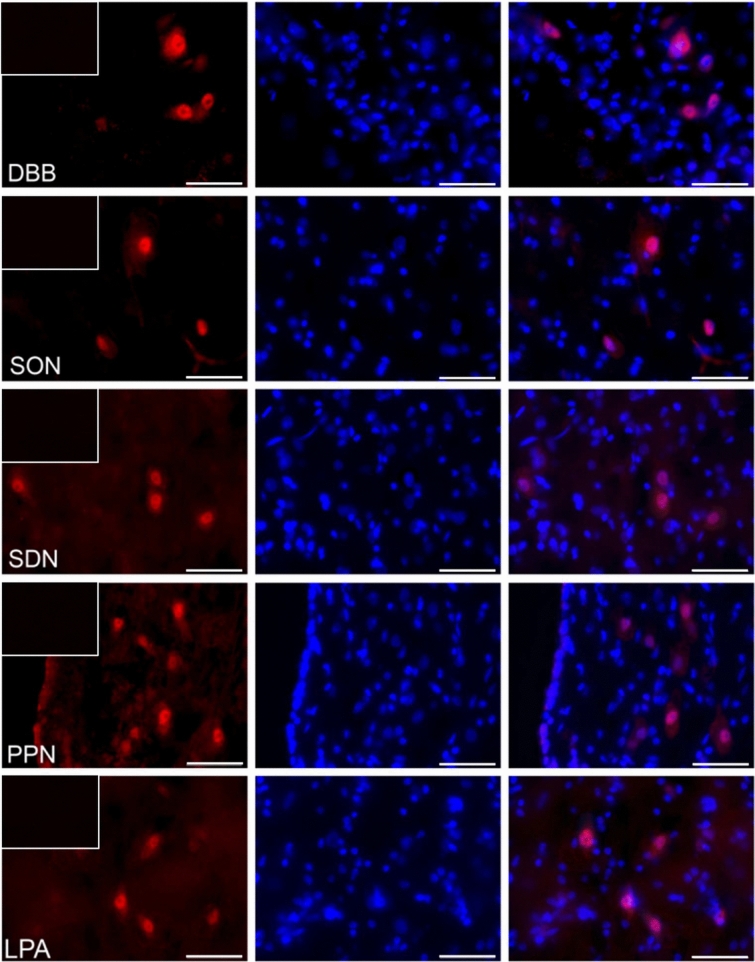

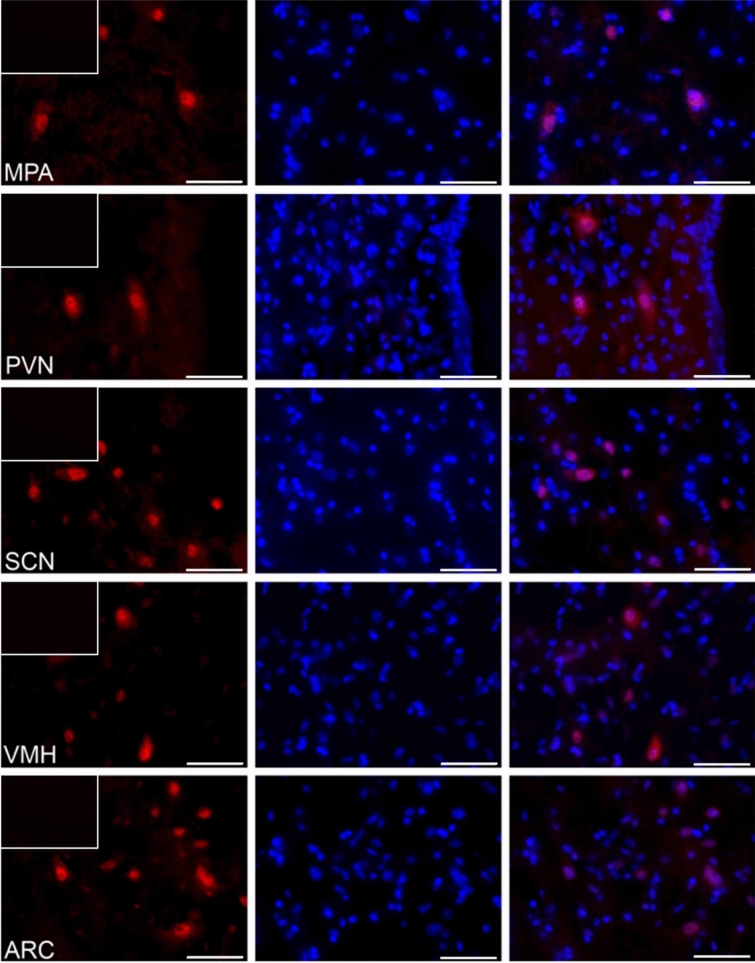
The visfatin localization in the porcine hypothalamus on days 15–16 of early pregnancy. Immunoreactivity of visfatin was determined by fluorescent immunohistochemistry. Columns from left: first—visfatin expression, visualized by Alexa Fluor 555 as red fluorescence; second—nuclei stained with DAPI, visualized as blue fluorescence; third—merged images of channels (Olympus BX51, Olympus Soft Imaging Solutions, Germany). In the upper left corner of each image in the first column—negative control, where the primary antibody were replaced with non-immunosera. Particular images indicate the immunolocalization of visfatin in the diagonal band of Broca (DBB), supraoptic nucleus (SON), preoptic area (SDN), periventricular nucleus (PPN), lateral (LPA) and medial preoptic area (MPA), paraventricular nucleus (PVN), suprachiasmatic nucleus (SCN), arcuate nucleus (ARC), as well as ventromedial nucleus (VMH). Immunofluorescent staining was repeated on three pigs. Scale bar: 50 μm.

The immunofluorescence staining has shown the presence of visfatin in some regions of the pig hypothalamus both during the estrous cycle (days 10–12—the mid-luteal phase; Fig. 1) as well as during early gestation (days 15–16 of pregnancy—the beginning of implantation; Fig. 2). Visfatin showed clear immunoreactivity in the supraoptic nucleus (SON), periventricular nucleus (PPN), sexually dimorphic nucleus of the preoptic area (SDN), paraventricular nucleus (PVN), lateral (LPA) and medial preoptic area (MPA), and additionally the diagonal band of Broca (DBB), a part of the basal forebrain and it was usually confined to the nucleus as well as neuroplasm of the cell bodies. In the suprachiasmatic nucleus (SCN), ventromedial nucleus (VMH), and arcuate nucleus (ARC), visfatin was located in the nucleus, sporadically was in cell neuroplasm. Staining was absent in all negative controls when the primary antibody was replaced with non-immunosera.

### Gene and protein expression of visfatin in the mediobasal hypothalamus

**Figure 3:**
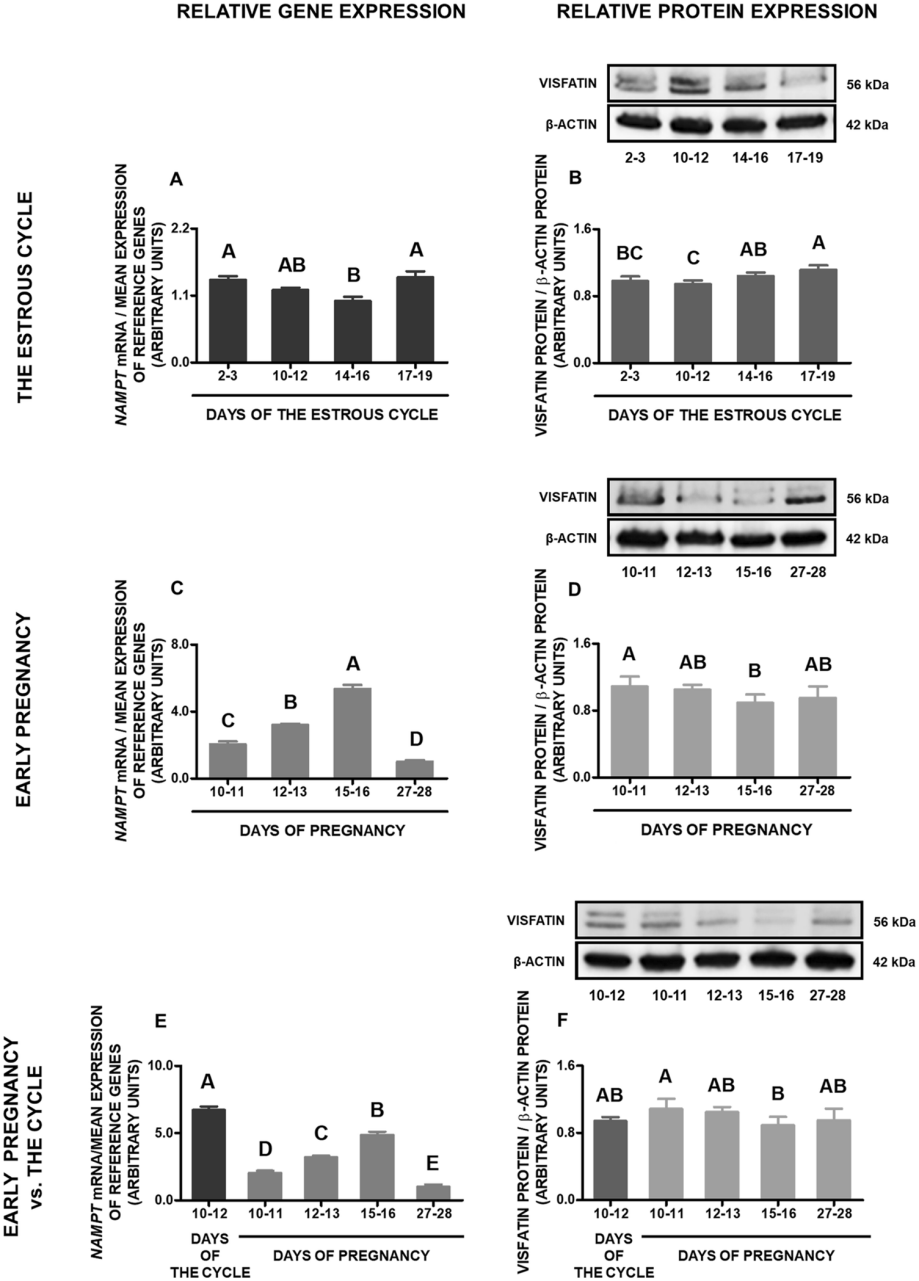
Visfatin gene and protein expression in the porcine mediobasal hypothalamus (MBH) during the estrous cycle and early pregnancy. Gene and protein expression of visfatin in the porcine MBH was determined during the estrous cycle on days: 2–3, 10–12, 14–16 and 17–19 (**A**,**B**), during early pregnancy on days: 10–11, 12–13, 15–16 and 27–28 (**C**,**D**) and compared between early pregnancy and days 10–12 of the estrous cycle (**E**,**F**). Gene expression was analyzed by qPCR. Protein expression was analyzed by Western blotting; upper panels: representative immunoblots; lower panels: densitometric analysis of visfatin protein relative to actin protein. Results are presented as means ± SEM (n = 5). Bars with different superscripts are significantly different (one-way ANOVA at *p* < 0.05 followed by Tuckey post hoc test at *p* < 0.05). Representative full length blots are attached as a Supplementary file 1.

During the estrous cycle, visfatin gene expression was higher on days 2–3 and 17–19 in comparison to days 14–16. In turn, the protein concentration of visfatin was the highest on days 17–19 compared to days 2 to 12 (*p* < 0.05; Fig. 3A,B; Supplementary file 1).

During early pregnancy, the lowest expression of visfatin gene was observed on days 27–28, however between days 10–11, 12–13, and 15–16 it was gradually increasing. Concerning the protein of visfatin, the highest concentration was observed on days 10–11 in relation to days 15–16 (*p* < 0.05; Fig. 3C,D; Supplementary file 1).

Comparing visfatin gene expression throughout the early pregnancy with days 10–12 of the estrous cycle, visfatin mRNA content during all periods of pregnancy was significantly lower. However, visfatin protein content in pregnant MBH samples was at a similar level as on days 10–12 of the cycle (*p* < 0.05; Fig. 3E,F; Supplementary file 1).

### Gene and protein expression of visfatin in the preoptic area

**Figure 4:**
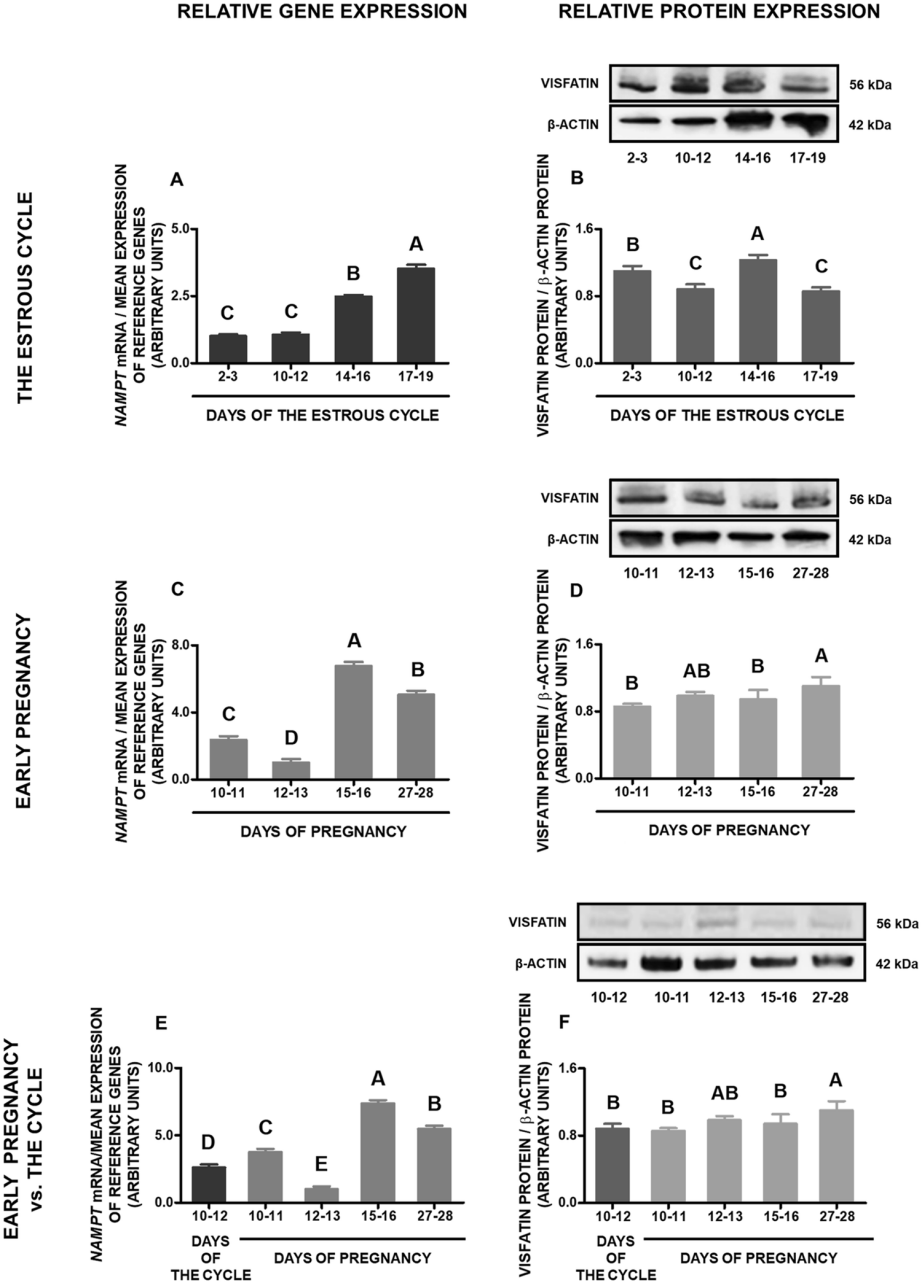
Visfatin gene and protein expression in the porcine preoptic area (POA) during the estrous cycle and early pregnancy. Gene and protein expression of visfatin in the porcine POA was determined during the estrous cycle on days: 2–3, 10–12, 14–16 and 17–19 (**A**,**B**), during early pregnancy on days: 10–11, 12–13, 15–16 and 27–28 (**C**,**D**) and compared between early pregnancy and days 10–12 of the estrous cycle (**E**,**F**). Gene expression was analyzed by qPCR. Protein expression was analyzed by Western blotting; upper panels: representative immunoblots; lower panels: densitometric analysis of visfatin protein relative to actin protein. Results are presented as means ± SEM (n = 5). Bars with different superscripts are significantly different (one-way ANOVA at *p* < 0.05 followed by Tuckey post hoc test at *p* < 0.05). Representative full length blots are attached as a Supplementary file 1.

During the estrous cycle, visfatin gene expression was the highest on days 17–19, lower on days 14–16, and the lowest on days 2–3 and 10–12. Concerning the protein level of visfatin, the highest content was noted on days 14–16, lower on days 2–3, and the lowest on days 10–12 and 17–19 (*p* < 0.05; Fig. 4A,B; Supplementary file 1).

During early pregnancy, the highest expression of visfatin gene was observed on days 15–16, and the lowest on days 12–13. In turn, the protein concentrations were the highest on days 27–28, and the lowest on days 10–11 and 15–16 of gestation (*p* < 0.05; Fig. 4C,D; Supplementary file 1).

Comparing the studied periods of early pregnancy with days 10–12 of the estrous cycle, we noted that visfatin gene expression on days 15–16, 27–28, and 10–11 of pregnancy was significantly enhanced, while the expression on days 12–13 of pregnancy was considerably lower compared to this phase of the estrous cycle. In turn, visfatin protein content on days 27–28 of pregnancy was essentially higher compared to the cycle, while during the other periods of pregnancy it was at a similar level as on days 10–12 of the cycle (*p* < 0.05; Fig. 4E,F; Supplementary file 1).

### Visfatin concentrations in the blood plasma

**Figure 5:**
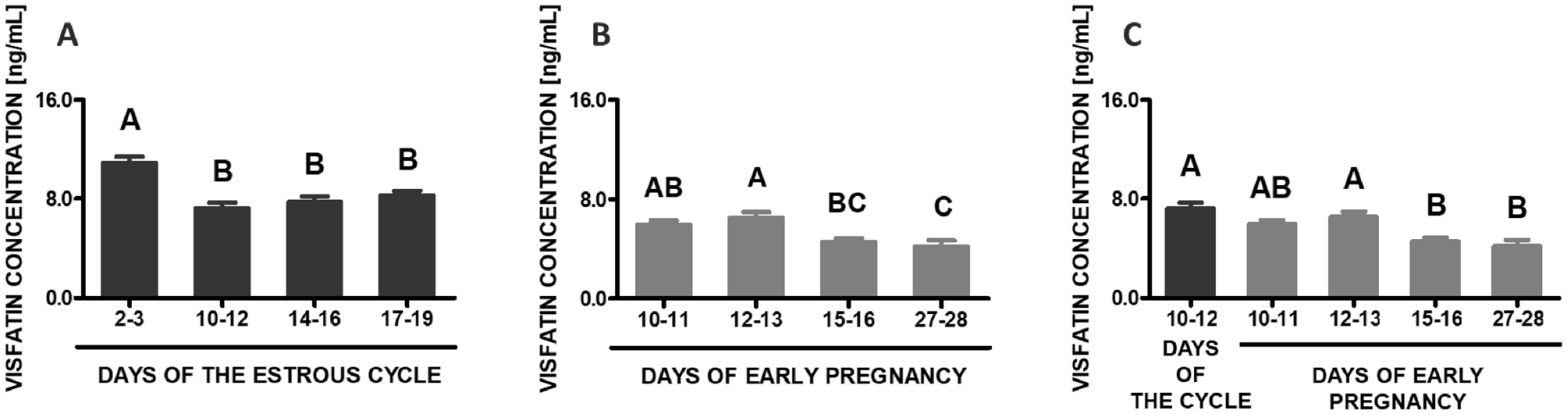
Visfatin concentration in the porcine blood plasma. Concentrations of visfatin in the porcine blood plasma was determined during the estrous cycle on days: 2–3, 10–12, 14–16 and 17–19 (**A**), during early pregnancy on days: 10–11, 12–13, 15–16 and 27–28 (**B**) and compared between early pregnancy and days 10–12 of the estrous cycle (**C**). The hormone content in blood plasma was evaluated using ELISA. Results are presented as means ± SEM (n = 5). Bars with different superscripts are significantly different (one-way ANOVA at *p* < 0.05 followed by Tuckey post hoc test at *p* < 0.05).

During the estrous cycle, a significantly higher concentration of visfatin was noted on days 2–3 in relation to other studied phases of the cycle (*p* < 0.05; Fig. 5A). During early pregnancy, the highest visfatin contents in the blood plasma were observed on days 12–13 compared to days 15 to 28 (*p* < 0.05; Fig. 5B), whereas the lowest on days 27–28 compared to days 10 to 13. The comparison of the studied stages of early pregnancy and days 10–12 of the estrous cycle has shown that the concentrations of visfatin in the blood plasma on days 15–16 and 27–28 were statistically lower than on days 10–12 of the cycle (*p* < 0.05; Fig. 5C).

## Discussion

The presented research was the first experiment to report the expression of visfatin gene and protein in the structures of the porcine hypothalamus responsible for GnRH production during the estrous cycle and early pregnancy. Based on our immunohistochemical analyses, visfatin was localized in both studied hypothalamic structures. These structures, including the medial preoptic area, diagonal band of Broca, lateral hypothalamic area, paraventricular nucleus, periventricular zone, suprachiasmatic nucleus and mediobasal hypothalamus, are also known as the place of GnRH neurons location in the pig brain^26,27^. Interestingly, visfatin was localized both in the cytoplasm of cells, what was earlier indicated in the mouse brain^28^, and the nucleus. It was suggested, based on the effect of cell cycle inhibition of 3T3-L1 preadipocytes on visfatin localization, that cell cycle arrest induces visfatin transport into the nucleus^29^. The expression pattern of visfatin gene in MBH and POA during the cycle and pregnancy was not parallel with the pattern of visfatin protein expression. Taking into account that in mammals the correlation between gene and protein expression frequently does not exceed 40%^30^, this observation is not surprising. The mentioned phenomenon can be attributed to transcriptional, post-transcriptional, and translational regulations and, as a consequence, discrepancies in mRNAs and proteins stability^31,32^.

In previous studies, visfatin gene expression was noticed in the mouse^4^ and chicken hypothalamus^30^, analyzed however as a whole, without division into particular hypothalamic structures. Visfatin expression was also found in the pituitary of male sheep^5^ and female mice^4^. In the mouse pituitary, visfatin was present mainly in gonadotroph cells, responsible for the follicle-stimulating hormone (FSH) and luteinizing hormone (LH) production^4^. To our best knowledge, this is the first study investigating visfatin gene and protein expression in the hypothalamus coupled with consideration of physiological status/hormonal milieu of animals characteristic for the estrous cycle and early pregnancy. The crucial hormonal factors related to the estrous cycle are progesterone (P_4_) and estradiol (E_2_). Most of them are of ovarian origin, but a minority is produced in the brain, including the hypothalamus, as neurosteroids^34^. Thus, the local concentration of steroids in the hypothalamus is a mixture of peripherally derived steroid hormones, converted peripheral steroids and neurosteroids^35,36^. Both neurons, oligodendrocytes and astrocytes are steroidogenically active cells and their primary steroidogenic product is neuroprogesterone^34^. It was indicated that astrocytes are the main source of the essential neuroprogesterone generated within the hypothalamic structures^37^. The expression pattern of visfatin gene and protein expression in MBH and POA is different throughout the estrous cycle and early pregnancy, which may suggest that the regulation of visfatin generation in both hypothalamic structures is also different. It seems possible that the observed in the MBH an increase of visfatin protein content on days 14–16 and 17–19 of the cycle is caused by the stimulatory action of E_2_, which plasma level is enhanced. In turn in the POA, the noticed suppressed visfatin expression on days 10–12 and 17–19 of the cycle can be coupled with P_4_ concentrations in the blood and hypothalamus. On days 10–12 of the porcine estrous cycle, plasma level of P_4_ is the highest, which may suggest that the hypothalamic concentration of this steroid is also enhanced, whereas on days 17–19 the same inhibitory effect is achieved by P_4_ produced mainly in the hypothalamus. Studies by Micevych et al.^38^ indicated that during proestrus the increased level of estrogens significantly induces neuroprogesterone synthesis in the hypothalamus. Circulating E_2_ also stimulated hypothalamic mRNA level and activity of 3β-hydroxysteroid dehydrogenase (3β-HSD), the key enzyme for P_4_ production. It was also suggested that locally synthesized hypothalamic neuroprogesterone, apart from its action via progesterone receptors (PR), may be 5α-reduced, converted to allopregnanolone, and bind to GABA_A_ receptors (GABA_A_R)^38^. It is postulated that this kind of GABA_A_R stimulation is involved in the induction of GnRH release^39^.

It is strongly suggested that the regulatory mechanism controlling visfatin expression is tissue- and species-specific^9,33^. The relationship between visfatin expression in particular tissues and the hormonal milieu of the organism is to a high degree unclear. On one hand, visfatin concentration in cerebrospinal fluid did not differ between men and women^40^. Similarly, there were no significant differences in visfatin mRNA expression in the mouse pituitary between the estrous and diestrus phases^4^. Generally, these reports indicate a lack of any dependence of visfatin generation on the hormonal status of the body, especially on the influence of sex steroid hormones. On the other hand, considerable evidence has accumulated to implicate the involvement of the hormonal environment in the control of visfatin expression. It has been indicated that the human maternal plasma concentration of visfatin was increased during pregnancy, especially between the two last and the first trimester^15^. One of the reasons for the observed increase may be many folds higher visfatin gene expression in omental fat of pregnant women at term compared to nonpregnant subjects^14^. Similarly in rats, visfatin mRNA content was elevated in the white fat on day 21 of pregnancy^41^. The results of our study also indicate the relationship of pregnancy with plasma visfatin level. Our observations are slightly different compared to the mentioned above studies—we noticed a decrease in plasma visfatin concentrations on days of implantation in relation to earlier days of pregnancy and days 10–12 of the estrous cycle. The reason for these differences may be, of course, the varied and species-dependent regulation of visfatin production throughout gestation, but the simplest explanation of the observed inconsistencies is the distinct (very early in the case of our studies) period of pregnancy taken into consideration in our and cited above work. Additional examples were obtained based on experiments with 3T3-L1 pre-adipocytes and adipocytes. Studies with pre-adipocytes have shown that visfatin gene expression decreased under influence of insulin, P_4_, and testosterone, and increased in response to dexamethasone^11^. In turn, treatment of 3T3-L1 adipocytes with GH, TNFα, cyclic adenosine monophosphate (cAMP), and beta-adrenergic agonists caused downregulation of the adipokine expression^13^, whereas the effect of sex hormones was insignificant^11^. More detailed studies concerning the possible influence of sex steroid hormones on visfatin expression in adipocytes indicated that estriol added to cell cultures increased visfatin mRNA concentration and the effect of P_4_ and E_2_ was negligible, but these three steroids used in combination essentially enhanced the gene expression^42^. Moreover, unlike the mouse pituitary, visfatin expression in the mouse uterus^43^ and ovary^43^ fluctuated during the estrous cycle. It has been shown that uterine visfatin protein concentration was increased in proestrus and metestrus, and decreased in estrus and diestrus. According to the authors, the reason for the observed changes can be the increased plasma estrogens levels in proestrus and their decrease in diestrus. Simultaneously, treatment with E_2_ of ovariectomized mice caused visfatin up-regulation, whereas P_4_ down-regulated visfatin expression. The co-treatment with both steroids enhanced visfatin protein content in the ovariectomized mouse uterus^44^. To a high degree similar visfatin protein expression was noticed in the mouse ovary, with the highest concentration observed in proestrus^43^. Taken together, the sum of our results and the results reported above suggest that expression of the visfatin gene may be hormonally regulated.

It has been suggested that besides local synthesis, an additional source of visfatin in the central nervous system may be transport across the blood–brain barrier. However, the concentrations of visfatin in human cerebrospinal fluid are at the level of approximately 10% of those in plasma. Moreover, visfatin levels in plasma and cerebrospinal fluid were negatively correlated as visfatin concentrations in cerebrospinal fluid decreased with increasing plasma visfatin contents in obese subjects^40^. Similarly, in our study, the concentration pattern of visfatin in both examined hypothalamic structures and in the plasma of pigs is completely different. It may suggest that visfatin transport across the blood–brain barrier, if there is, does not play a major role, and the local hypothalamic regulation of visfatin production is autonomic and distinct in relation to other tissues, especially the adipose tissue which seems to be the main source of the adipokine.

The visfatin role in the hypothalamus is to a high degree unknown. As previously mentioned, visfatin is both an intracellular enzyme and a secreted hormone. Especially its localization in cell nuclei seems to be intriguing and may suggest, based on the data provided by Svoboda et al.^29^, that NAMPT, through stimulation of NAD biosynthesis and sirtuins activation, takes part in the regulation of DNA repair, chromatin structure, transcription, replication, telomerase length and circadian rhythm. It has been indicated day-night differences of visfatin expression in the ovine pars tuberalis and increased visfatin expression, under melatonin influence, in this structure connecting the hypothalamus and pituitary^5^. It seems that links between energy sensing and NAD cycle, and the regulation of circadian clock function exist also in other parts of the central nervous system^45^. Visfatin, acting at the hypothalamic level as an energy metabolism sensor, may be involved in the control of food intake, principally fasting- and ghrelin-induced food intake^46^. Other studies, using visfatin knockin mice, indicated enhanced NAD^+^ levels in multiple tissues, including the hypothalamus, and an improvement in physical activity, sleep quality, and cognitive functions^47^. Nevertheless, visfatin effect on pituitary secretory functions remains unknown. Its expression in the structures responsible for GnRH production may suggest that visfatin is also engaged in the control of GnRH generation. The intracerebroventricular injection of visfatin stimulated GnRH-I gene expression in chicks^48^. The adipokine also enhanced Fos gene expression in the mouse hypothalamic explants^49^, a marker of neuroendocrine cell activation^50^. This hypothesis, however, should be confirmed in further detailed studies.

Similarly, the role of visfatin in pregnancy is poorly understood. It seems that the adipokine is mainly involved in the control of maternal and fetal metabolism, both at the central and peripheral level^16^, regulating in this way reproductive functions according to energy balance fluctuations. It has been indicated that the circulating levels of visfatin were positively correlated with the intrauterine fetal growth throughout pregnancy^51^. Moreover, visfatin has been linked to homeostasis regulation of amniotic fluid through stimulation of vascular endothelial growth factor receptor 2 expression in the placenta^52^, inhibition of contractions of the pregnant rat and human myometrium^53^, and initiation of labor via activation of pro-inflammatory cytokine release^54^.

Summarizing, visfatin expression in the porcine hypothalamic structures responsible for GnRH production implies its autocrine/paracrine influence on GnRH synthesis and confirms the potential role of visfatin as a neuromodulator of reproductive functions. The variations in the expression levels of visfatin noticed during the estrous cycle and early pregnancy may suggest the effect of steroid hormones, both peripheral and hypothalamic origin. It cannot be ruled out either, that the influence of steroids is achieved through their action on hypothalamic neural systems, like GABAergic, dopaminergic, serotoninergic systems engaged in the control of GnRH production. Further research focused on steroid hormones and their receptors concentrations in particular hypothalamic nuclei involved in GnRH synthesis is also required.

## Methods

### Animals and tissues collection

Tissue samples were harvested from animals intended for commercial slaughter and meat processing, and the collected tissues were an abattoir by-product. Animal slaughter, tissue collection and transportation of biological material to the laboratory were carried out in accordance with the Polish Act on the protection of animals used for scientific or educational purposes of the 15th of January 2015 (Journal of Laws Dz.U. 2015 No. item 266) as well as directive 2010/63/EU of the European Parliament of the 22nd of September 2010 on the protection of animals used for scientific purposes, so this study did not require the consent of the competent ethics committee for animal experiments. Mature cross-breed gilts (Large White × Polish Landrace) aged 7–8 months and weighing 140–150 kg, obtained from the private breeding farm (L. Wisniewski breeding farm, Krolikowo, Poland), were used in the study. Animals' diet was balanced (crude protein, metabolizable energy, exogenous amino acids and minerals) following the Polish nutritional standards for domestic pigs, with access to fresh water ad libitum. To investigate the visfatin expression, a total of forty animals were divided into eight experimental groups (n = 5 per group), as follows: days 2–3 (early-luteal phase, presence of corpora haemorrhagica), 10–12 (mid-luteal phase, the phase when the corpus luteum activity is the highest and similar to that noted during pregnancy), 14–16 (late-luteal phase, the phase of luteolysis) and 17–19 (follicular phase) of the estrous cycle, as well as days 10–11 (migration of the embryos within the uterus), 12–13 (maternal recognition of pregnancy), 15–16 (beginning of implantation) and 27–28 (end of implantation) of pregnancy. Gilts were monitored daily for estrus behavior in the presence of a boar. The day of the onset of the second estrus was marked as day 0 of the estrous cycle. The phase of the estrous cycle was also confirmed based on the morphology of ovaries^55^. In the case of pregnant pigs, natural insemination was performed on days 1–2 of the estrous cycle. In the case of days 15–16 and 27–28 of pregnancy, the stage of pregnancy was also confirmed by the presence and morphology of conceptuses/trophoblasts^56^. Within a few minutes after slaughter, blood samples were collected into heparinized tubes and centrifuged at 2500 × *g* for 15 min at 4 °C. The obtained plasma was stored at − 20 °C for further measurement. Subsequently, the hypothalamus was removed and the MBH and POA were excised as previously described^57^. The mediobasal hypothalamus was defined as a block of tissue bounded rostrally by the optic chiasma, caudally by the mammillary body, laterally by the hypothalamic sulci and dorsally by a 5 mm-deep cut. The preoptic area was limited rostrally approximately 5 mm anterior to the optic chiasma and caudally by the rostral border of the MBH. The mediobasal hypothalamus and POA obtained on days 15–16 of pregnancy and 10–12 of the cycle were divided into two parts, from which one half was intended for immunofluorescent staining (placed in 4% buffered paraformaldehyde; pH = 7.4, 4 °C), and the other half was frozen in liquid nitrogen and stored at − 80 °C until processing for RNA and protein analyzis.

### Radioimmunological measurement of plasma progesterone and estradiol levels

To link the levels of the most important sex steroid hormones to the phase of the estrous cycle and confirm the correctness of the evaluation of the cycle phase, the levels of P_4_ and E_2_ were determined according to the methods described by Ciereszko et al.^58^ and Hotchkiss et al.^59^, respectively. The extraction efficiency for P_4_ was 86.45±0.75 and for E_2_ 88.59±1.29%. Cross-reactivities of the antisera against P_4_ and E_2_ have been reported by Dziadkowiec et al.^60^ and Szafranska et al.^61^, respectively. The validity of both assays was confirmed by the parallelism between the standard curves and a series of dilutions of the samples. The sensitivities of the assays for P_4_ were 2 pg and for E_2_ 0.5 pg per tube. The intra-assay coefficients of variations of P_4_ and E_2_ assays were 7.3±0.84 and 7.9± 0.87%, respectively (the inter-assay coefficients of variations were not determined because the assays were done in one assay). The plasma level of P_4_ on days 2–3, 10–12, 14–16, and 17–19 was as follows: 1759.2±85.45^B^, 10308.8±1556.8^A^, 3174.55±150.31^B^ and 2292.2±276.86^B^ pg/ml, while the serum concentrations of E_2_ on the same days were 62.41±5.94^B^, 66.78±4.27^B^, 64.64±2.75^B^ and 80.25±4.58^A^ pg/ml, respectively. All values correspond with earlier published data describing sex steroid concentrations in porcine plasma during the estrous cycle^62^.

### The analyzis of visfatin localization in the porcine hypothalamus using fluorescent immunohistochemistry

To verify visfatin occurrence in the porcine hypothalamus, qualitative immunohistochemical analysis was carried out in three cyclic (days 10–12 of the estrous cycle) and three pregnant animals (days 15–16 of pregnancy). The tissue blocks were fixed by immersion for 36 h in 4% buffered paraformaldehyde (pH = 7.4; 4 °C). Following fixation, the brains were washed in 0.1 M phosphate-buffered saline (PBS) and then cryoprotected for 3–6 days in graded solutions (19% and 30%) of sucrose (Sigma-Aldrich, USA) at 4 °C until fully infiltrated. The tissues were frozen and cut into 12 μm thick cryostat coronal sections and stored at − 80 °C. Localization of the hypothalamic nuclei was calculated based on Felix et al.^63^. Frozen brain sections were processed for routine single-immunofluorescence labeling. All steps of the staining procedures were conducted in humid chambers at room temperature. The sections were air-dried for 30 min, washed 3 times in PBS, and incubated for 1 h with blocking buffer (0.1 M PBS, 10% normal horse serum, 0.01% bovine serum albumin (BSA), 1% Tween, 0.05% thimerosal, 0.01% NaN_3_). Then, the sections were incubated overnight with rabbit polyclonal antibodies against visfatin (1:600; cat.-no. ab233294, Abcam, UK). Following subsequent rinsing in PBS (3 × 15 min), the sections were incubated (1 h) with the Alexa Fluor 555 donkey anti-rabbit antibodies (1:1000; cat.-no. A-31572, Thermo Fisher Scientific, USA). After that, the slides were washed in PBS and coverslipped with histology mounting medium Fluoroshield with DAPI (Sigma-Aldrich, USA) for nuclear counterstaining. The sections were analyzed with an Olympus BX51 microscope equipped with an Olympus XM10 digital camera (Tokyo, Japan). Images were acquired with cellSens Dimension 1.7 Image Processing software (Olympus Soft Imaging Solutions, Münster, Germany). Negative controls, i.e. omission and replacement of primary antisera by non-immunosera, were applied to test antibody and method specificity. Lack of any immunoreaction indicated specificity.

### The analyzis of NAMPT gene expression in the porcine hypothalamus using quantitative real-time PCR

Total RNA was isolated using the TRI reagent (Sigma-Aldrich, USA) according to the manufacturer's instructions. The quantity and quality of the isolated RNA were determined spectrophotometrically (Infinite M200 Pro, Tecan, Switzerland). One microgram of RNA was reverse transcribed into cDNA using the Omniscript RT Kit (Qiagen, Germany) with 0.5 μg oligo(dt)_15_ primers (Roche, Germany) in a total volume of 20 μl at 37 °C for 1 h and then terminated at 93 °C for 5 min. Quantitative real-time PCR (qPCR) analyzis was performed using an AriaMx Real-Time PCR System (Agilent Technologies, USA) with Power SYBR Green Master Mix (Applied Biosystems Inc., USA), as described previously^64^. Specific primer pairs used to amplify parts of *NAMPT*, *UBC* (ubiquitin C), and *18sRNA* (18S ribosomal RNA) genes are detailed in Table 1. The *UBC* and *18sRNA* were used as reference genes. Preliminary studies confirmed that their expressions were stable in both studied tissues during all studied stages of the estrous cycle and early pregnancy (similar Ct values without statistically significant differences). The qPCR reaction mixtures at the final volume of 20 μl contained 20 ng of cDNA, the appropriate forward and reverse primers at the concentrations detailed in Table 1, 12.5 μl of Power SYBR Green PCR Master Mix (Applied Biosystems, USA) and RNase free water. The qPCR conditions are detailed in Table 1. Negative controls contained RNase free water instead of cDNA. All reactions were amplified in duplicates. The analyzis of the melting curve was used to confirm the specificity of amplification. The relative gene expression level was calculated using the comparative cycle threshold method (∆∆Ct)^65^ and normalized using the geometrical means of reference genes expression levels of *UBC* and *18sRNA*. The Ct values for all non-template controls were under the detection threshold.

**Table 1:** Characteristics of primers used in the study.

Gene symbol	Primers sequences	Reaction Conditions	Number of cycles	Primer (nM)	Target sequence accession number	References
*NAMPT*	F: 5′-CCAGTTGCTGATCCCAACAAA-3′ R: 5′-AAATTCCCTCCTGGTGTCCTATG-3′	Activation: 50 °C, 2 min Activation: 95 °C, 10 min Denaturation: 95 °C, 15 s Annealing: 60 °C, 1 min	40	300	XM_003132281.5	^68^
*UBC*	F: 5′-GGAGGAATCTACTGGGGCGG-3′ R: 5′ -CAGAAGAAACGCAGGCAAACT-3′	Activation: 95 °C, 10 min Denaturation: 95 °C, 15 s Annealing: 60 °C, 1 min Elongation: 70 °C, 1 min	40	400	XM_003483411.3	^67^
*18sRNS*	F: 5′-TCCAATGGATCCTCGCGGAA-3′ R: 5′-GGCTACCACATCCAAGGAAG-3′	Activation: 95 °C, 10 min Denaturation: 95 °C, 15 s Annealing: 60 °C, 1 min Elongation: 70 °C, 1 min	40	400	AY265350.1	^67^

*NAMPT* visfatin*, UBC* Ubiquitin C*, 18sRNA* 18S ribosomal RNA*,* *F* forward, *R* reverse.

### The analyzis of visfatin protein expression in the porcine hypothalamus using Western blot

Western blotting and quantification were performed as previously described^66^. About 30-mg pieces of porcine MBHs and POAs were homogenized in 500 µl of T-PER Tissue Protein Extraction Reagent (Thermo Fisher Scientific, USA). Equal amounts of the lysate (50 μg protein/sample) were separated in Mini-Protean TGX System Precast Protein Gels (Bio-Rad Laboratories Inc., USA) and then transferred to Trans-Blot Turbo Mini PVDF Transfer Packs (Bio-Rad Laboratories Inc., USA). The membranes were blocked for 1 h in 0.02 M Tris-buffered saline containing 5% BSA and 0.1% Tween 20, then incubated overnight at 4˚C with primary anti-visfatin antibody (cat.-no. ab233294; Abcam, UK) diluted at 1:700. Subsequently, the membranes were washed with TBST (Tris-buffered saline containing 0.1% Tween 20 Detergent) and incubated for 1 h at room temperature with a horseradish peroxidase-conjugated antibody (cat.-no. 7074; Cell Signaling Technology, USA) diluted at 1:1000. The anti-β-actin antibodies (cat.-no. A5316; Sigma-Aldrich, USA) diluted at 1:5000 were used as a loading control. The reference protein was detected on the same membranes as the target protein. After the densitometry analysis of the visfatin protein abundance, the membranes were stripped, incubated with the appropriate antibodies, and then used for the densitometry analysis of the protein abundance of β-actin. Signals were detected by chemiluminescence using the Western blotting Luminol Reagent (Advansta Inc., USA), and visualized using the Chemidoc XRS + System (BioRad Laboratories Inc., USA). All visible bands were quantified using a densitometer and ImageJ software (US National Institutes of Health, USA).

### The analyzis of visfatin concentration in the porcine blood plasma using enzyme-linked immunosorbent assay

The concentrations of visfatin protein in plasma were determined using a commercial ELISA kit (cat.-no. MBS736963, MyBioSource, USA) according to the manufacturer’s protocol. Absorbance values were measured at 450 nm using an Infinite M200 Pro reader with Tecan i-control software (Tecan, Switzerland). The data were linearized by plotting the log of visfatin concentrations versus the log of the optical density and the best fit line was determined by regression analyzis. The intra-assay coefficient of variation of the ELISA assay for visfatin was 5.29%.

### Data analyzis

Statistical analyzis was performed as previously described^67^. All experimental data were presented as means ± standard error of the mean (SEM) from five different observations. Differences between groups were analyzed by one-way ANOVA followed by Tukey’s honest significant difference post hoc test. All data were tested for the assumptions of normality (Shapiro–Wilk test) and homogeneity of variances (Levene's test). All ANOVA assumptions have been fulfilled. Statistical analyses were performed using Statistica software (StatSoft Inc., Tulsa, USA). Values for *p* < 0.05 were considered as statistically significant and identified by different letters.

### Ethics declarations and approvals for animal experiments

Tissue samples were harvested from animals intended for commercial slaughter and meat processing, and the collected tissues were an abattoir by-product. Animal slaughter, tissue collection, and transportation of biological materials to the laboratory were carried out in accordance with the Polish Act on the protection of animals used for scientific or educational purposes of the 15th of January 2015 (Journal of Laws Dz.U. 2015 No. item 266) as well as the directive 2010/63/EU of the European Parliament of the 22nd of September 2010 on the protection of animals used for scientific purposes, so this study did not require the consent of the competent ethics committee for animal experiments.

## Supplementary Information

Supplementary Information
