# Supplementary material for: Plasma level and expression of visfatin in the porcine hypothalamus during the estrous cycle and early pregnancy
Source: Sci Rep. 2021 Apr 22;11:8698. doi: 10.1038/s41598-021-88103-z (PMC8062436; doi:10.1038/s41598-021-88103-z)

Plasma level and expression of visfatin in the porcine hypothalamus during the estrous cycle and early pregnancy

Tadeusz Kaminski, Marta Kiezun, Ewa Zaobidna, Kamil Dobrzyn, Barbara Wasilewska, Ewa Mlyczynska, Edyta Rytelewska, Katarzyna Kisielewska, Marlena Gudelska, Kinga Bors, Grzegorz Kopij, Karolina Szymanska, Barbara Kaminska, Agnieszka Rak and Nina Smolinska

MBH

VISFATIN

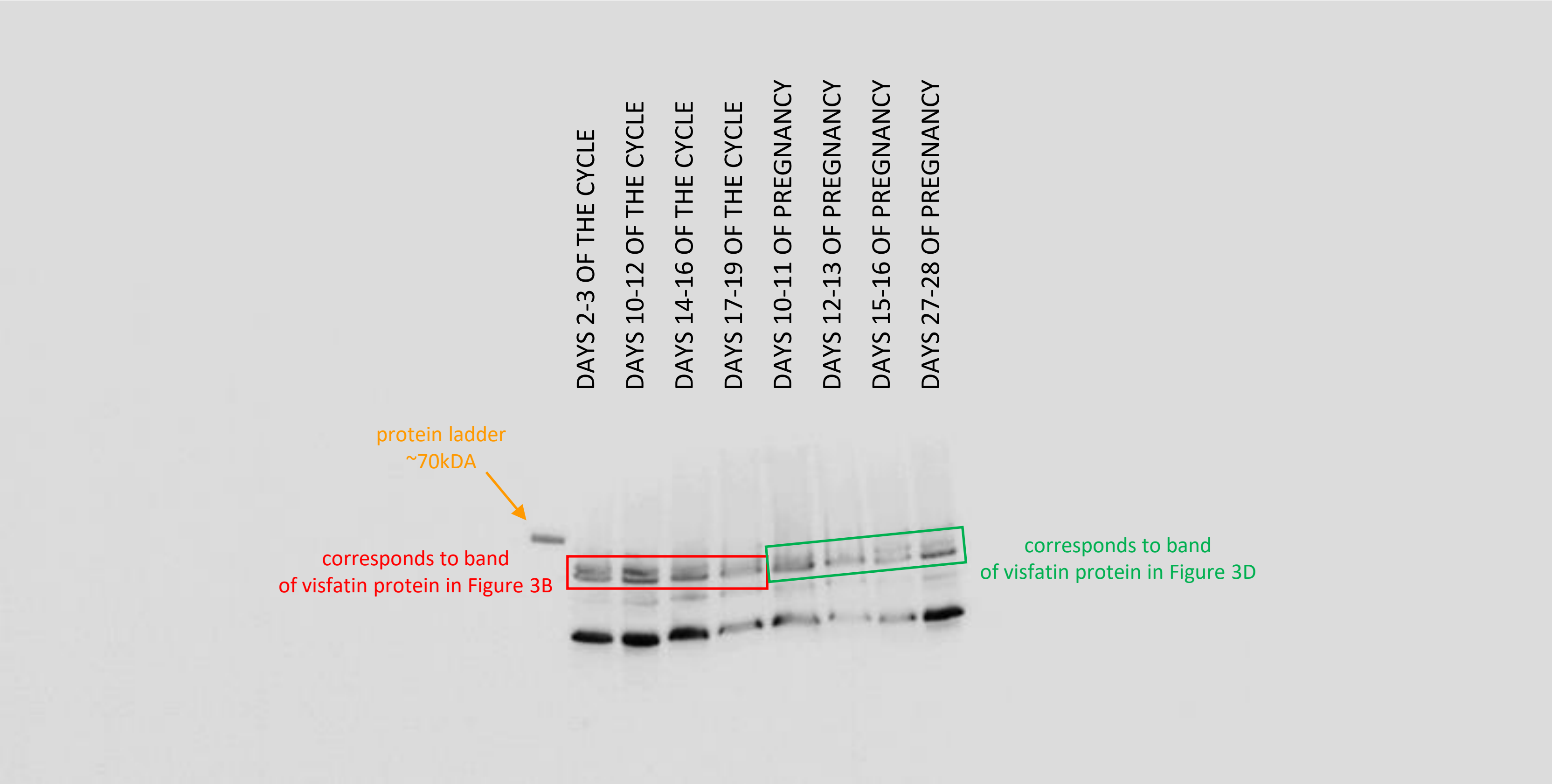

Plasma level and expression of visfatin in the porcine hypothalamus during the estrous cycle and early pregnancy

Tadeusz Kaminski, Marta Kiezun, Ewa Zaobidna, Kamil Dobrzyn, Barbara Wasilewska, Ewa Mlyczynska, Edyta Rytelewska, Katarzyna Kisielewska, Marlena Gudelska, Kinga Bors, Grzegorz Kopij, Karolina Szymanska, Barbara Kaminska, Agnieszka Rak and Nina Smolinska

MBH

ACTIN

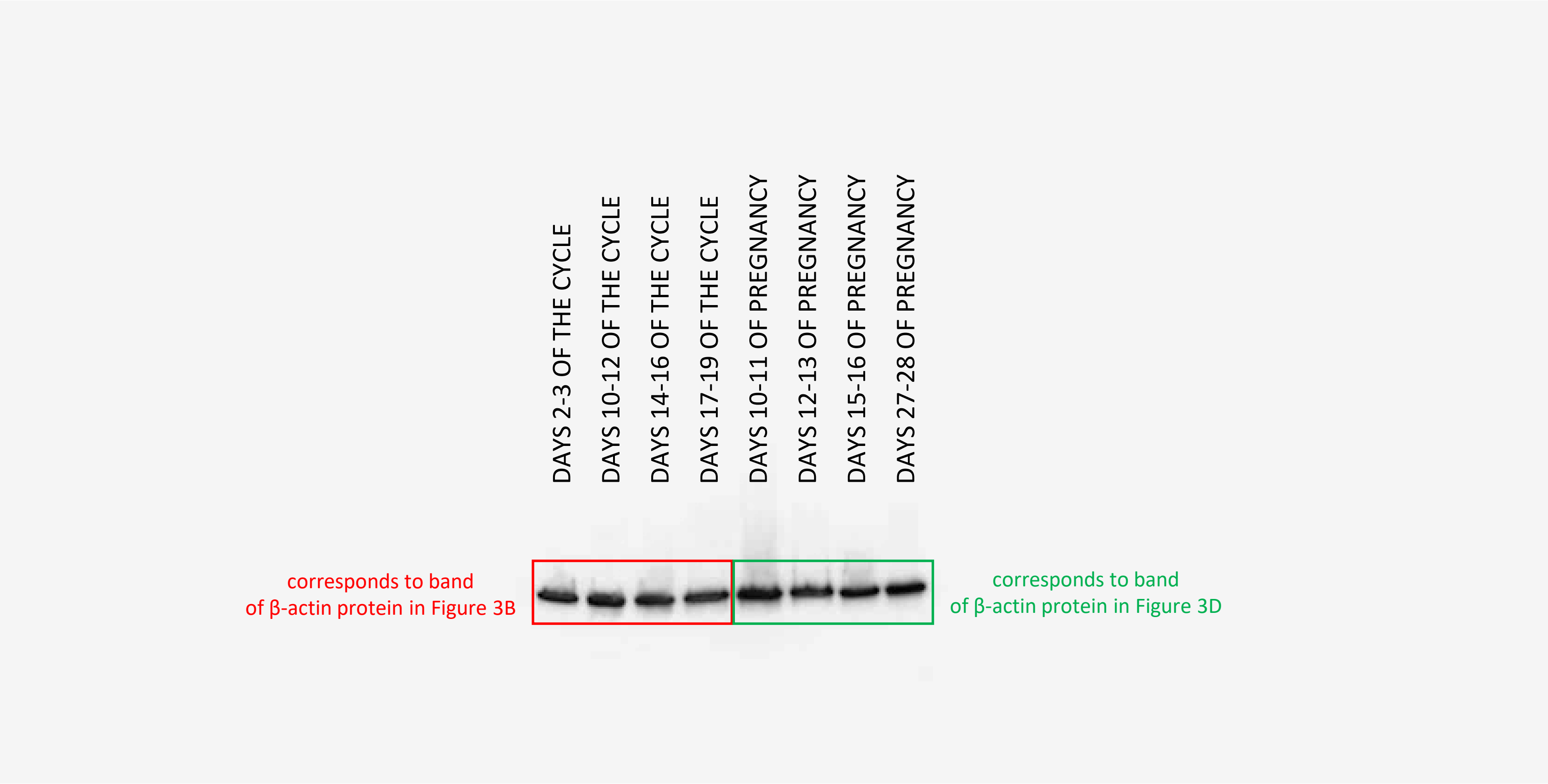

Plasma level and expression of visfatin in the porcine hypothalamus during the estrous cycle and early pregnancy

Tadeusz Kaminski, Marta Kiezun, Ewa Zaobidna, Kamil Dobrzyn, Barbara Wasilewska, Ewa Mlyczynska, Edyta Rytelewska, Katarzyna Kisielewska, Marlena Gudelska, Kinga Bors, Grzegorz Kopij, Karolina Szymanska, Barbara Kaminska, Agnieszka Rak and Nina Smolinska

MBH

VISFATIN

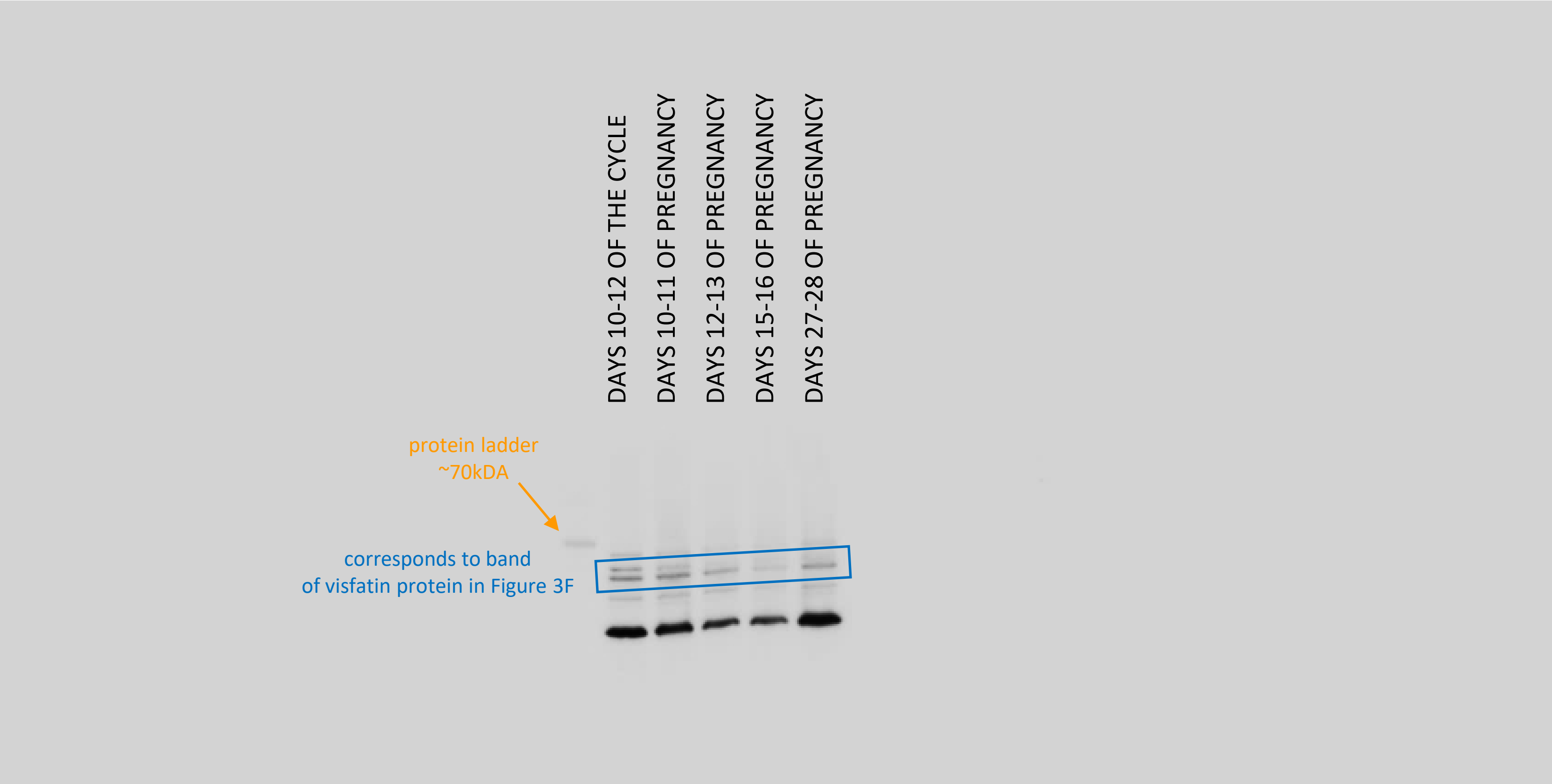

Plasma level and expression of visfatin in the porcine hypothalamus during the estrous cycle and early pregnancy

Tadeusz Kaminski, Marta Kiezun, Ewa Zaobidna, Kamil Dobrzyn, Barbara Wasilewska, Ewa Mlyczynska, Edyta Rytelewska, Katarzyna Kisielewska, Marlena Gudelska, Kinga Bors, Grzegorz Kopij, Karolina Szymanska, Barbara Kaminska, Agnieszka Rak and Nina Smolinska

MBH

ACTIN

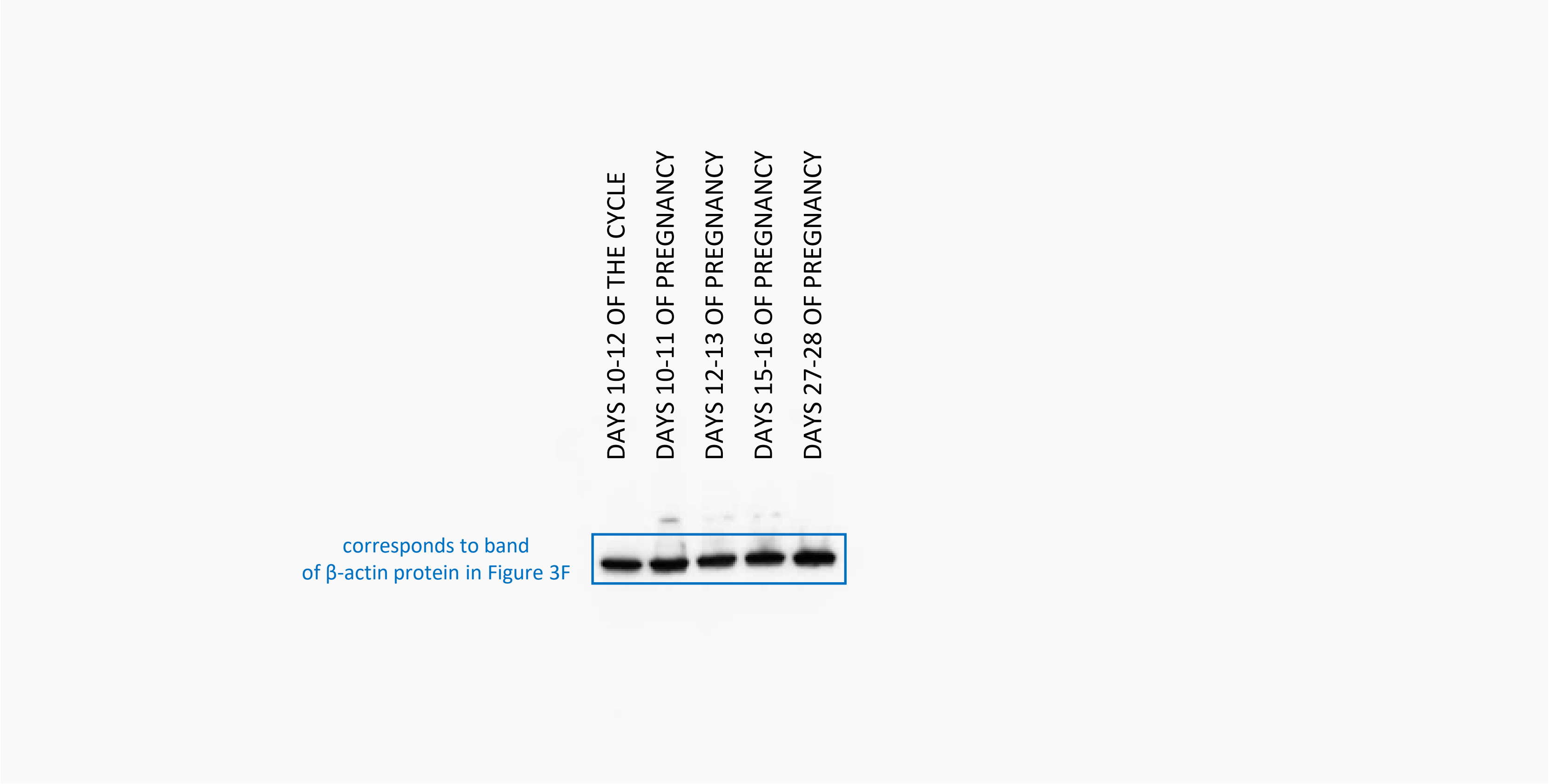

Plasma level and expression of visfatin in the porcine hypothalamus during the estrous cycle and early pregnancy

Tadeusz Kaminski, Marta Kiezun, Ewa Zaobidna, Kamil Dobrzyn, Barbara Wasilewska, Ewa Mlyczynska, Edyta Rytelewska, Katarzyna Kisielewska, Marlena Gudelska, Kinga Bors, Grzegorz Kopij, Karolina Szymanska, Barbara Kaminska, Agnieszka Rak and Nina Smolinska

POA

VISFATIN

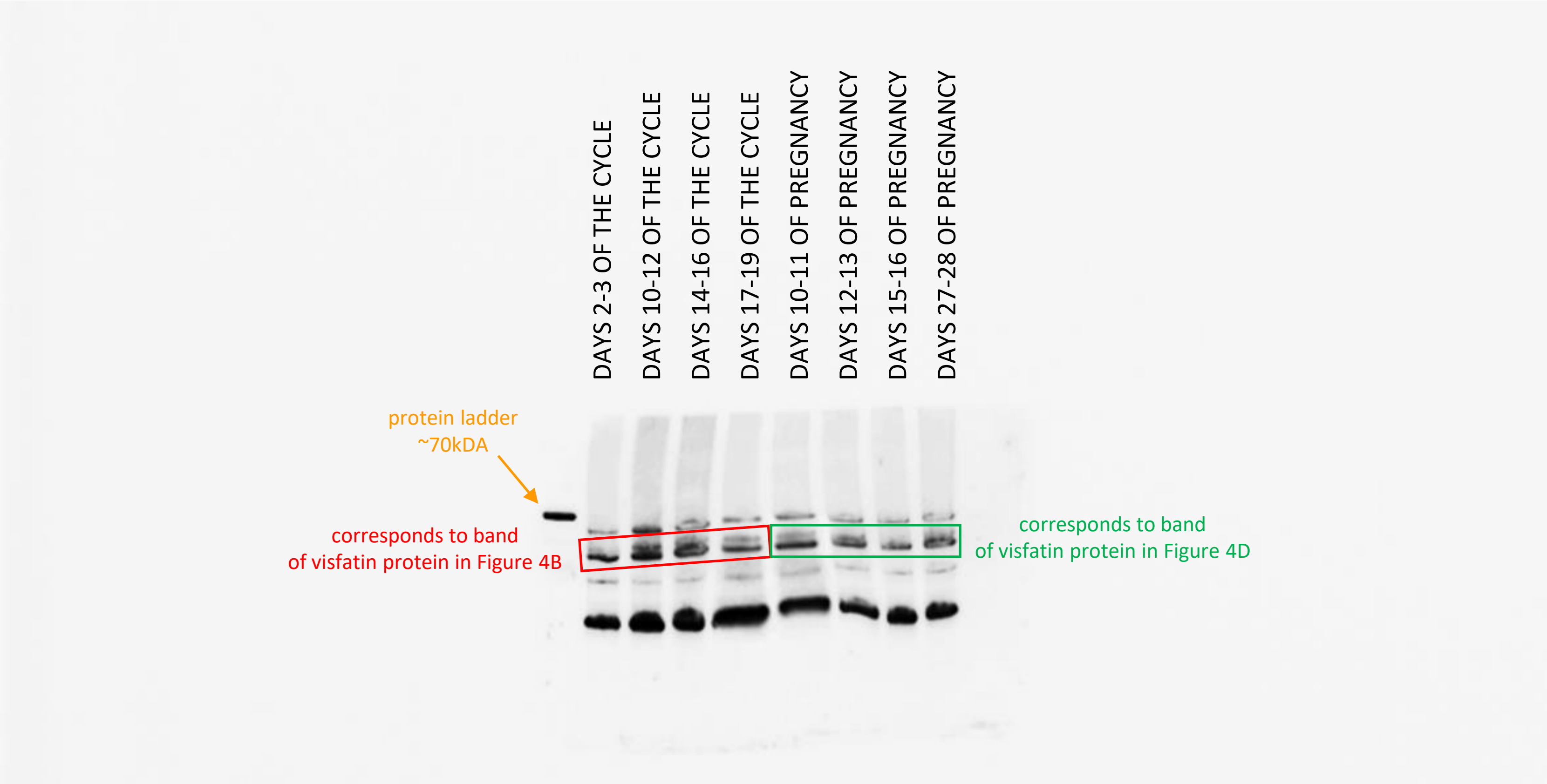

Plasma level and expression of visfatin in the porcine hypothalamus during the estrous cycle and early pregnancy

Tadeusz Kaminski, Marta Kiezun, Ewa Zaobidna, Kamil Dobrzyn, Barbara Wasilewska, Ewa Mlyczynska, Edyta Rytelewska, Katarzyna Kisielewska, Marlena Gudelska, Kinga Bors, Grzegorz Kopij, Karolina Szymanska, Barbara Kaminska, Agnieszka Rak and Nina Smolinska

POA

ACTIN

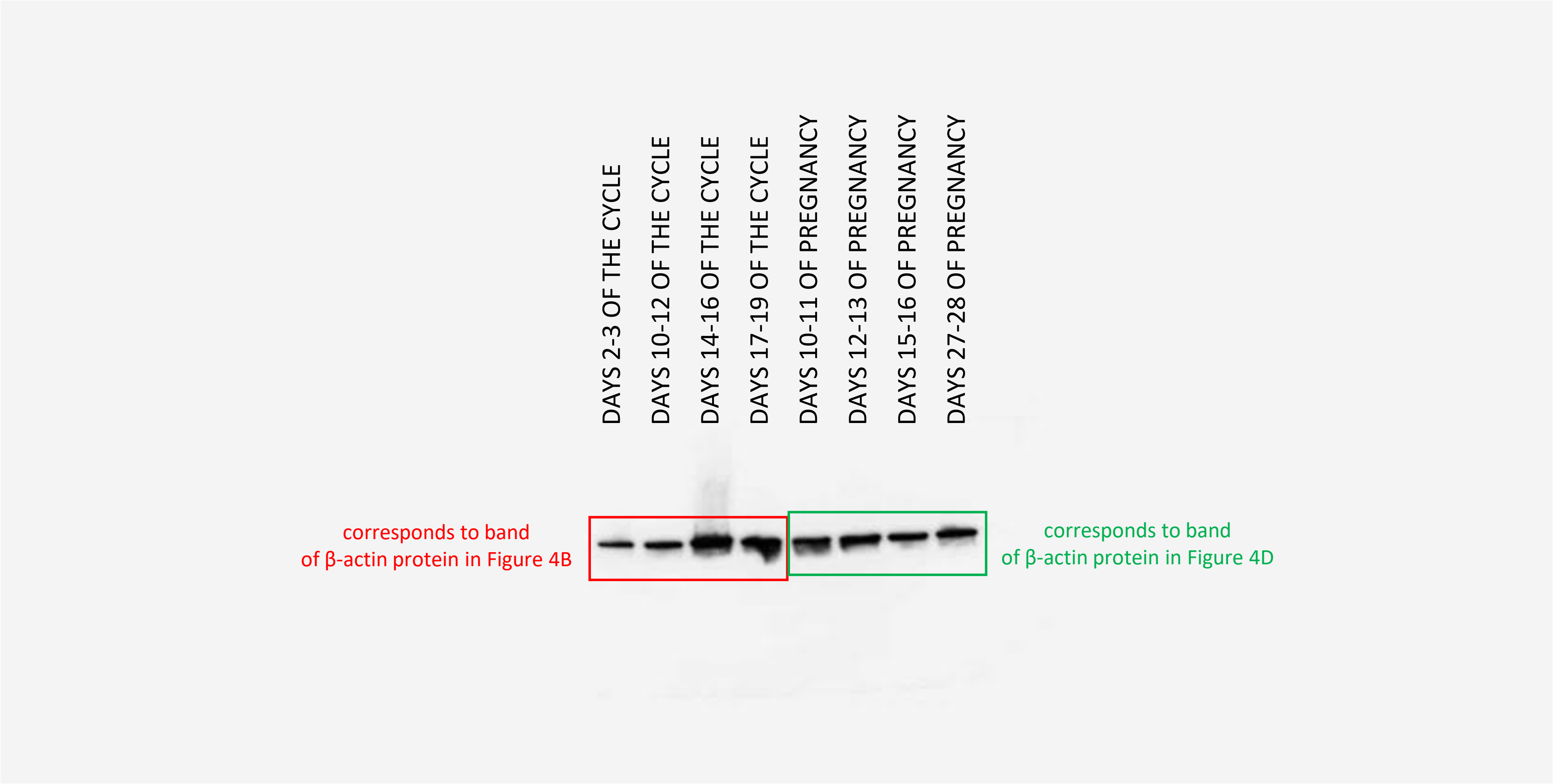

Plasma level and expression of visfatin in the porcine hypothalamus during the estrous cycle and early pregnancy

Tadeusz Kaminski, Marta Kiezun, Ewa Zaobidna, Kamil Dobrzyn, Barbara Wasilewska, Ewa Mlyczynska, Edyta Rytelewska, Katarzyna Kisielewska, Marlena Gudelska, Kinga Bors, Grzegorz Kopij, Karolina Szymanska, Barbara Kaminska, Agnieszka Rak and Nina Smolinska

POA

VISFATIN

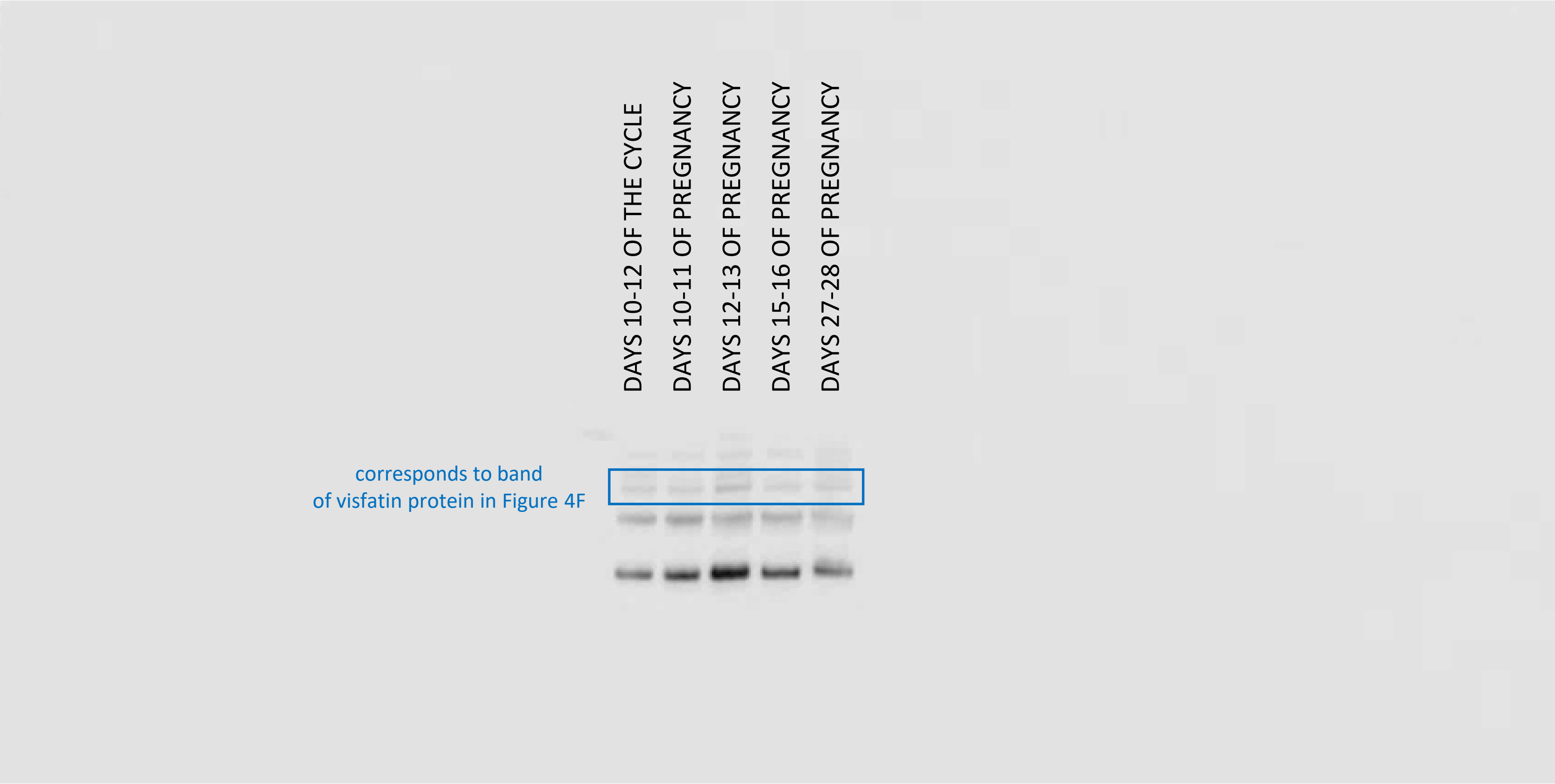

Plasma level and expression of visfatin in the porcine hypothalamus during the estrous cycle and early pregnancy

Tadeusz Kaminski, Marta Kiezun, Ewa Zaobidna, Kamil Dobrzyn, Barbara Wasilewska, Ewa Mlyczynska, Edyta Rytelewska, Katarzyna Kisielewska, Marlena Gudelska, Kinga Bors, Grzegorz Kopij, Karolina Szymanska, Barbara Kaminska, Agnieszka Rak and Nina Smolinska

POA

ACTIN

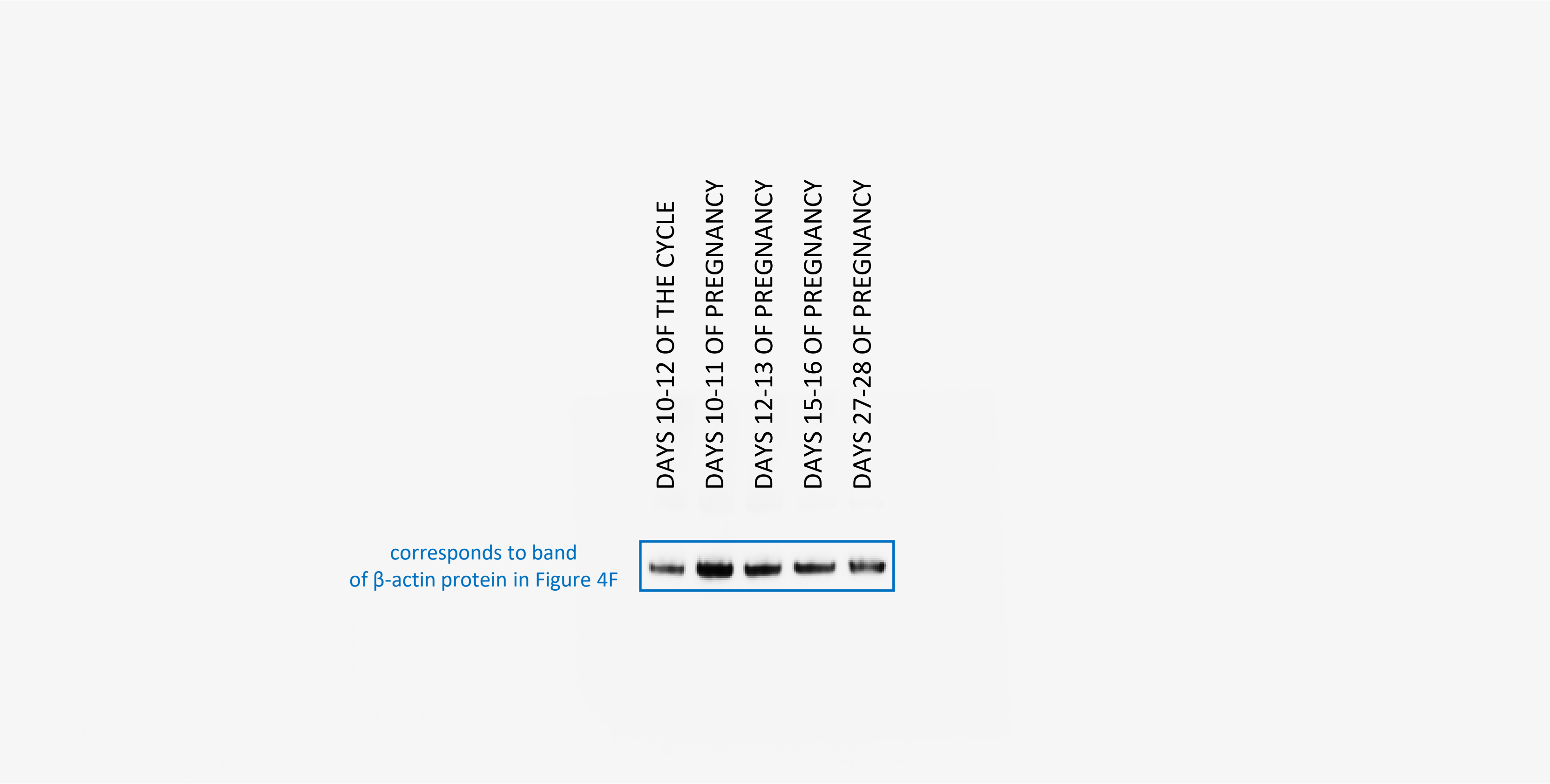

Supplement: Supplementary file 1 — Supplementary Information [file 41598_2021_88103_MOESM1_ESM.pdf]
